# Pazopanib with Topotecan weekly for patients with platinum-resistant or intermediate-sensitive recurrent ovarian cancer: results of a multicentre, open label phase I/II study (TOPAZ)

**DOI:** 10.1007/s00432-023-04647-9

**Published:** 2023-03-31

**Authors:** Radoslav Chekerov, Tjadina Arndt, Klaus Pietzner, Ulrich Canzler, Pauline Wimberger, Hans-Georg Strauß, Sven Mahner, Linn Woelber, Nikolaus de Gregorio, Gertraud Stocker, Ekkehard von Abel, Tanja Neunhoeffer, Antje Kristina Belau, Alexander Mustea, Isil Yalinkaya, Elena Ioana Braicu, Rolf Richter, Jalid Sehouli, for the NOGGO ovarian cancer study group

**Affiliations:** 1 Department of Gynaecology with Center for Oncological Surgery, Charité-Universitätsmedizin Berlin, corporate member of Freie Universität Berlin, Humboldt-Universität zu Berlin and Berlin Institute of Health, Berlin, Germany; 2 Department of Gynaecology and Obstetrics TU Dresden and National Center for Tumor Diseases (NCT/UCC), Dresden, Germany; 3 Department of Gynaecology Universitätsklinik und Poliklinik, Martin-Luther University, Halle-Wittenberg, Germany; 4 Department of Obstetrics and Gynaecology, University Hospital, Ludwig-Maximilian-University, Munich, Germany; 5 Department of Gynaecology, University Medical Center Hamburg-Eppendorf, Hamburg, Germany; 6 Department of Obstetrics and Gynaecology, SLK Klinikum Heilbronn, Heilbronn, Germany; 7 Medical Department, University Cancer Center Leipzig (UCCL), University Medical Center, Leipzig, Germany; 8 Department of Obstetrics and Gynaecology, Stauferklinikum Schwäbisch Gmünd, Schwäbisch Gmünd, Germany; 9 Department of Gynaecology, Helios Dr. Horst-Schmidt-Kliniken, Wiesbaden, Germany; 10 Department of Gynaecology, University of Greifswald, Greifswald, Germany; 11 Department of Gynaecology and Gynaecological Oncology, University of Bonn, Bonn, Germany

**Keywords:** Platinum-resistant ovarian cancer, Topotecan, Pazopanib

## Abstract

**Purpose:**

Pazopanib has promising antiangiogenetic activity in solid cancers. The investigator-initiated phase I/II trial evaluated the combination of Topotecan with Pazopanib in platinum-resistant or intermediate-sensitive recurrent ovarian cancer (ROC).

**Methods:**

Patients (≥ 18 years) with first or second recurrence were enrolled in this multicentre open-label trial. Phase I analysed Topotecan 4 mg/m^2^ (day 1, 8, 15, ever 28 days) for six cycles to identify the maximum tolerated dose (MTD) of Pazopanib added in a dose-escalating scheme with 400 mg starting dose. The phase II analysed safety and efficacy aspects. For all patients with clinical remission a maintenance with Pazopanib until progression was allowed. This trial is registered with ClinicalTrials.gov, number NCT 01600573.

**Results:**

Between June 2012 and February 2017, 11 patients were enrolled in the phase I, and 50 patients in the phase II study. The MTD of Pazopanib was determined by 400 mg/daily. Haematological and liver toxicities determined the dose limiting toxicities (DLT) and the most common grade 3–4 adverse events: leucopenia (25%), neutropenia (22%), thrombocytopenia (19%), accumulation of cholestatic (20%) and hepatocellular damage (15%), which often caused dose modifications, but no new life-threatening events. Overall response was 16% and clinical benefit rate 68%. Median progression-free survival (PFS) was 3.5 months (95% CI 2.0—5.0). Due to early progression only 20% of the patients were able to start with maintenance treatment.

**Conclusion:**

The combination of pazopanib and weekly topotecan is feasible, resulting in a manageable haematological and liver toxicity, but despite its encouraging response rate, was not associated with a significant survival benefit.

## Introduction

Epithelial ovarian cancer remains the gynaecological malignancy with the highest mortality being the seventh most common cancer in women worldwide (Sung et al. 2021). Despite the progress in surgical and systemic treatment with implementation of anti-angiogenetic and poly (ADP-ribose) polymerase (PARP) inhibitors, more than 75% of all patients will progress with development of multidrug-resistance disease (Colombo 2019; Bois et al. 2009). Recurrent ovarian cancer becomes a palliative character with generally less standardised treatment options dependent on the treatment-free interval (TFI) to the last platinum chemotherapy and genetic features like BRCA mutation status. Especially for patients with short TFI of less than 6 months, where platinum seems not to be the best treatment option, the chance of remission remains low, which results in a particularly poor prognosis (Bois et al. 2009; Wilson et al. 2017). Current treatment strategies are still based on platinum free monotherapies, with a commonly short treatment horizon due to development of early disease progression (Hanker et al. 2012; Sehouli et al. 2008). Thus, there is still an urgent need for implementation of new therapeutic options.

In patients with platinum-resistant ovarian cancer, the randomized phase 3 AURELIA trial, demonstrated for the first time an improvement of progression-free survival (PFS) incorporating bevacizumab, a VEGF-inhibitor, to single-agent chemotherapy selected by investigator’s choice (weekly paclitaxel, pegylated liposomal doxorubicin [PLD], or topotecan), while the effect on overall survival remained not significant (Pujade-Lauraine et al. 2014). Within the topotecan cohort the hazard ratio for PFS was 0.32 (95% CI 0.21–0.49) supporting the combination with bevacizumab (median 5.8 vs. 2.1 months with topotecan alone) and with objective response rate of 17% (0% for topotecan alone) (Pujade-Lauraine et al. 2014). Based on these findings a new era in the treatment of recurrent ovarian cancer was opened (Chase et al. 2017). Other new classes of antiangiogenic agents with proven drug activity are the tyrosine kinase inhibitors (TKI) like pazopanib, sorafenib, nintedanib and cediranib, as also the angiopoietin inhibitor trebananib, most of them improving PFS, although overall survival (OS) remained not beneficial (Bois et al. 2014; Bois et al. 2013; Pignata et al. 2015; Chekerov et al. 2018; Ledermann et al. 2016; Monk et al. 2014). Moreover, in platinum-resistant ovarian cancer, the randomized double-blind phase 2 TRIAS trial, assessed sorafenib, a multi TKI, in the combination with topotecan (1.25 mg/m^2^ d1-5) (Chekerov et al. 2018). Till now this is the only trial demonstrating a statistically and clinically significant improvement of PFS for topotecan plus sorafenib with a median of 6.7 months (95% CI 5.8–7.6) vs. 4.5 months for placebo (95% 3.7–5.0, HR 0.6, *p* = 0.0018). An additional OS-effect was shown for the combination with sorafenib (median 17.1 months, 95% CI 12.5–21.7) versus 10.1 months for the placebo arm (95% CI 7.7–12.5, HR 0.65, p = 0.017), demonstrating the essential role of targeting angiogenesis as an innovative treatment strategy (Chekerov et al. 2018).

Pazopanib, a non-selective oral multi-kinase inhibitor limits tumour growth by targeting angiogenesis, resulted by hypoxemia of the tumour microenvironment, via inhibition of different enzymes of the VEGF-receptor family (Schutz et al. 2011; Hamberg et al. 2010). Pazopanib is approved by the US Food and Drug Administration and European Medicines Agency for treatment of renal cell carcinoma and soft-tissue sarcoma (Boudou-Rouquette et al. 2016; Graaf et al. 2012). Pazopanib demonstrated promising single-agent activity in patients with solid cancers, also in recurrent ovarian cancer with moderate toxicity profiles similar to other small-molecule inhibitors (hypertension, liver dysfunction, haematological effects) (Friedlander et al. 2010; Hurwitz et al. 2009). The randomized phase 3 AGO-Ovar16 trial analysed the incorporation of pazopanib (800 mg/once daily) as a maintenance approach in patients with advanced ovarian cancer, who had not progressed after first-line chemotherapy. This study demonstrated with a 5.6 month prolongation in PFS a strong effect of pazopanib on maintenance treatment (17.9 months versus 12.3 for placebo, HR 0.77, 95% CI 0.64—0.91, *p* = 0.0021), however, this approach did not influenced OS (Bois et al. 2014; Vergote et al. 2019). In preclinical ovarian cancer models pazopanib showed significant antitumor and antiangiogenic activity in the combination with metronomic topotecan (Merritt et al. 2010). In patients with platinum-resistant ovarian cancer two trials analysed the impact of pazopanib. The randomized MITO-11 trial combined paclitaxel with pazopanib and showed a significantly longer PFS compared to paclitaxel alone (median 6.35 months, 95% CI 5.36–11,02 vs. 3.49 months, 95% CI 2.01–5.66, HR 0.42, *p* = 0.0002), but a simultaneous improvement of 5.4 months for OS was not significant (Pignata et al. 2015). In the randomized GINECO TAPAZ study 116 patients with early recurrence after bevacizumab maintenance did not show any survival difference for the combination of weekly paclitaxel and pazopanib (600–800 mg daily) compared to paclitaxel alone. Both studies demonstrated significantly more grade 3/4 adverse events (AE’s) and more toxicity-related treatment discontinuations (Pignata et al. 2015; Joly et al. 2022).

Topotecan, the well-known topoisomerase I inhibitor is one of the established treatment options for recurrent platinum-resistant ovarian carcinoma (Sehouli et al. 2008; Edwards et al. 2015). The randomized phase 2 TOWER study analysed the weekly administration of topotecan compared with the conventional 5-day regime (Sehouli et al. 2011). This study identified a better tolerability for the weekly schedule with lower rates of haematological toxicity, but also slightly lower effectiveness in terms of response rates and PFS, which nevertheless makes topotecan weekly a feasible and viable treatment option for patients with recurrent ovarian cancer (Sehouli et al. 2011). These scientific experience and promising study data encouraged us to study pazopanib in combination with topotecan, in a simultaneous schedule followed by maintenance treatment to determine feasibility, safety and first activity effects in recurrent ovarian cancer.

## Patients and methods

### Study design and objectives

TOPAZ was a multicentre, single arm, prospective, phase I/II study, performed by academic researchers of the North-Eastern German Society of Gynaecologic Oncology (NOGGO) ovarian cancer study group. The phase-I part was conducted within a 21-month recruitment period (increased with 6 months in the first protocol amendment on December 12, 2012 and with 9 months by the second protocol amendment on Mai 27, 2013) at two German gynaecological oncology centres—the Charité University Hospital Berlin and the University Hospital Leipzig. In the phase II conducted within a recruitment period of 30 months (increased with 6 months in the fourth protocol amendment on March 3, 2015, and with 12 months by the fifth protocol amendment on April 13, 2016) participated seven additional German gynaecological oncology centres.

The phase I as dose-escalation study had the primary objective to determine the maximum tolerated dose (MTD) of pazopanib by identification of the dose-limiting toxicity (DLT) of the combination of weekly topotecan and pazopanib, while the phase II aimed to analyse toxicity profiles and safety of the combination with a primary endpoint on progression-free survival (PFS) as described per RECIST. Overall survival, response rate (i.e. complete response (CR) and partial response (PR)) and clinical benefit rate (i.e. the sum of CR, PR and stable disease (SD)) evaluated according RECIST-criteria and CA-125 according to Gynecologic Cancer InterGroup (GCIG) criteria, duration of response, time to progression, quality of life as obtained by EORTC-QLQ C30 and OVAR 28 questionnaires were defined as secondary study objectives of the phase II. During the recruiting period the protocol was approved with amendments documenting the changes of pazopanib manufacturer and the marketing authorisation holder for topotecan.

### Patient population

Women age ≥ 18 years with histologically confirmed ovarian, peritoneal or fallopian tube cancer who had progressed during platinum therapy (platinum refractory) or within 6 months after completing primary or secondary platinum-containing therapy (platinum resistant) or within 12 months after primary platinum-containing therapy (intermediate platinum-sensitive) were eligible for study treatment. Additional inclusion criteria were measurable disease as described per RECIST 1.1 or elevated cancer antigen (CA)- 125 as described by GCIG criteria; ability to swallow and retain oral medication; Eastern Cooperative Oncology Group (ECOG) status 0 or 1; life expectancy of ≥ 12 weeks; adequate bone marrow, liver, renal and cardiac function (defined as haemoglobin ≥ 9 g/dl, absolute neutrophile count ≥ 1500 cells per μl, platelet count ≥ 100 cells per μl, prothrombin time, international normalised ratio or partial thromboplastin time ≤ 1.2 × upper limit of normal (ULN), total bilirubin ≤ 1.5 × ULN, alanine aminotransferase and aspartate aminotransferase ≤ 2.5 × ULN, calculated creatinine clearance ≥ 50 ml/min or serum creatinine ≤ 1.5 mg/dl). Women of childbearing potential had to agree to adequate contraception throughout the study.

Patients were ineligible if they had received more than two previous chemotherapies, previous topotecan at any time or had surgery, radiotherapy or concurrent antineoplastic therapy up to two weeks before inclusion into study. Other exclusion criteria were prior diagnosis of any malignancy ≤ 5 years before study entry (except successfully treated in situ carcinomas or skin basal cell carcinoma), brain metastasis, any kind of gastrointestinal disease that could interfere with the study medication, grade 3 or 4 diarrhoea, ulcer or bleeding diathesis, uncontrolled arterial hypertension, clinically relevant heart condition or a long QT syndrome > 450 ms, endobronchial lesions with vessel infiltration or fracture.

Ethical review committees approved the study protocol, amendments, and other relevant study documentation. The study was conducted according to the Declaration of Helsinki (1996), the International Conference on Harmonization Good Clinical Practice recommendations, and provisions of the German Medicines Act. All patients provided written informed consent before study start. Alcedis as independent clinical research institute was responsible for data monitoring.

### Treatment plan

The backbone of the chemotherapy for both phase I and II parts of the study was weekly topotecan 4 mg/m^2^ administered as an intravenous infusion over 30 min on days 1, 8 and 15, repeated every 28 days, for up to maximum of 6 cycles. In the phase I dose-escalation part pazopanib was given orally every day with a starting dose of 400 mg (dose level 1) and in absence of DLT escalated to dose of 600 mg (level 2) and 800 mg (level 3). To determine the DLT of pazopanib at least 3 patients have to be assessable at each of the planned dose levels. If any patient developed DLT during the first treatment cycle, the number of patients in this dose level will be increased to six. If there were no DLT’s, the MTD was defined as the highest dose level in which none or only one out of six patients experienced DLT in the first cycle. In case of ≥ 2 DLT at the evaluated dose level, the protocol stipulated all subsequent patients to be treated in the previous dose level, while dose re-escalation was not allowed. Drop-outs for other reasons than toxicity were replaced.

Patients in the phase II received pazopanib in the dose as determined by the phase I trial. In case of complete or partial response or stable disease the pazopanib therapy could be maintained until development of unacceptable toxicity or tumour progression according the RECIST criteria. Patients who discontinued drug therapy early were observed for 12 months from end of chemotherapy. Concomitant therapies with supportive or other medications as transfusions of fresh frozen plasma and blood components were allowed to treat emesis, pain, infections and other complications of the malignancy and treatment.

### Safety and efficacy assessments

Toxicity was assessed for each cycle, graded according to the NCI-Common Terminology Criteria for Adverse Events (CTCAE) version 4.0. Dose-limiting toxicity were defined as: grade 3 or 4 non-hematologic toxicity, grade 3 thrombocytopenia combined with bleeding (removed with third protocol amendment, March 26, 2014) or grade 4 thrombocytopenia, grade 4 neutropenia lasting ≥ 7 days or febrile neutropenia defined as ANC < 1000/µl concurrent with fever, any grade 2 and more toxicity of cycle 1 other than nausea, vomiting, rash, alopecia or anaemia, that persisted over 35 days.

At study baseline gynaecological and physical examination, weight control, vital signs, electrocardiogram (ECG), ECOG, laboratory parameters (haemoglobin, leucocytes, neutrophils, platelets, partial thromboplastin time (PTT), prothrombin time [international normalised ratio] (PT-INR), aspartate aminotransferase, alanine aminotransferase, gamma-glutamyl transferase, bilirubin, alkaline phosphatase, urea, albumin, creatine [and creatine clearance if applicable], TSH, free T4, sodium, magnesium, and potassium) and quality of life questionnaires EORTC-QLQ C30 and OVAR 28 were obtained. Further laboratory evaluation (haemoglobin, leucocytes, neutrophils, platelets, PTT and PT-INR) was performed weekly during the phase I and the first cycle of the phase II study. Prior to every further treatment cycle ECOG and physical examination with addition of cardiac monitoring per ECG every 4 weeks were performed. During pazopanib maintenance the safety monitoring was continued every four weeks, with assessment of liver parameters (aspartate aminotransferase, alanine aminotransferase, gamma-glutamyl transferase, bilirubin) biweekly (weeks 5, 7, 9) and from the first follow-up every three weeks of pazopanib intake. During the follow-up assessment of survival, physical status, tumour response, ECOG and quality of life were obtained every three months.

At baseline radiological assessment CT or MRI (physician’s choice) of measurable and non-measurable tumour lesions (≤ 28 days prior study enrolment) and CA-125 monitoring were achieved. For response assessment, radiological evaluation (RECIST) and CA-125 measurements (GCIG criteria) were done every 12 weeks from the start of treatment and during the maintenance phase. Tumour response had to be confirmed by a second examination, no independent review of outcome was necessary.

### Statistical analysis

The phase I of this study was designed to ensure an adequate assessment of the toxicity of topotecan with pazopanib and to determine MTD and DLT, based on the continuous reassessment method. A maximum of 18 patients may be enrolled in three pre-planned dose escalation levels. After each dose level step of this part of the trial an interim safety analysis was performed.

Based on the published results of the phase 3 TOWER study, the assumed median PFS of the conventional topotecan monotherapy was 4.4 months (Sehouli et al. 2011). The statistical hypothesis of the phase II part assumed, that the PFS at 6 months will be expected by 50% and under the treatment with topotecan and pazopanib the median PFS will increase to 6 months for patients with recurrent, pre-treated ovarian cancer. This was considered as clinically significant and seems to be a realistic goal. The width of the 95% confidence interval for the estimated PFS at 6 months would be about 14 percentage points (absolute size) when 50 patients are recruited into this study part.

Median PFS, as primary objective of the phase II, was defined as the time (months) from start of the first treatment cycle until disease progression (PD) or death, or date of last follow-up. Duration of response was defined as time from first assessment of response until PD or death, and the time to progression was the time from start of the first therapy cycle until PD is observed.

All analyses of therapeutic efficacy will be summarised, in addition to a qualitative review of safety, tolerability and quality of life conducted in an exploratory fashion. Results are presented as raw numbers, rates, medians with 95% CI or ranges. Event related data like progression free survival, time to progression, duration of response and overall survival time was analysed by the non-parametric Kaplan–Meier method. All statistical calculations were implemented by SAS (Statistical Analysis System, SAS Institute, North Carolina, USA). This trial is registered with ClinicalTrials.gov, number NCT01600573.

## Results

### Patient characteristics and disposition

**Fig. 1:**
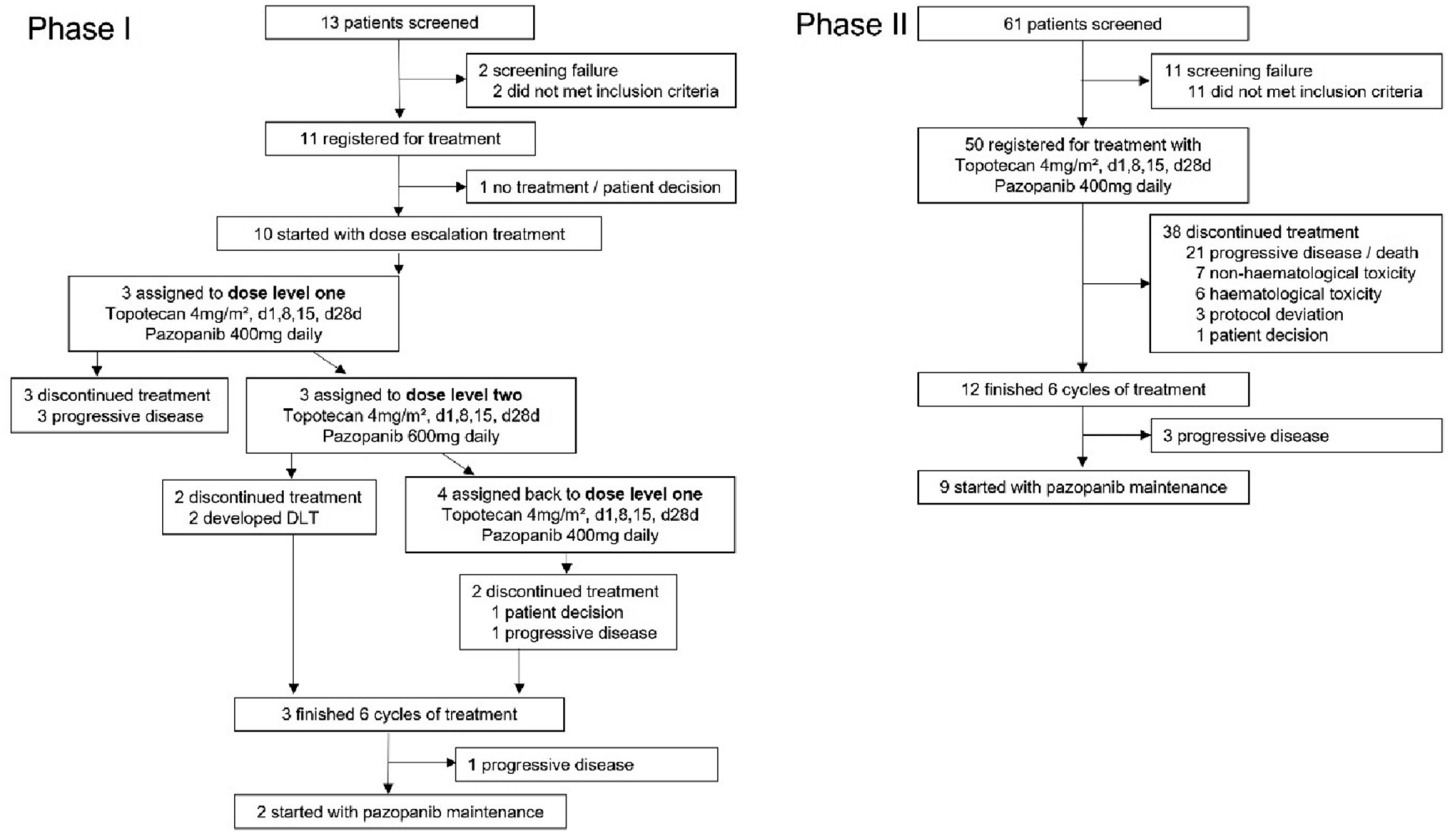
Patient recruitment and treatment within the TOPAZ phase I/II trial

In total 11 patients were registered into the phase I of the TOPAZ study, of whom ten received study treatment (Fig. 1). The phase II single arm study enrolled additional 50 patients from nine German study centres (Fig. 1). The main baseline characteristics were well balanced between the phase I and II cohorts with median age of 55 years (range 30–76) (Table 1). Despite recruiting patients with predominantly platinum-refractory or resistant first (21%) and second (57%) recurrent disease, most participants were of good performance status and with predominantly high-grade serous histology with ovarian (88%), fallopian tube (10%) or primary peritoneal cancer (2%) origin.

**Table 1:** Baseline clinical characteristics of the patient’s cohorts

Patient’s characteristics, *n* (%)		Phase I	Phase II
Registered		11	50
Age	Median	55	56
	Range	36—72	30—76
ECOG - Performance status	0	7 (64)	26 (52)
	1	4 (36)	24 (48)
Histology	Serous	9 (82)	40 (80)
	Other	2 (18)	10 (20)
FIGO	1	0	5 (10)
	2	1 (9)	3 (6)
	3	4 (36)	25 (50)
	4	1 (9)	12 (24)
	Unknown	5 (46)	5 (10)
Grading	G1	1 (9)	4 (8)
	G2	2 (18)	7 (14)
	G3	7 (64)	27 (54)
	Unknown	1 (9)	12 (24)
Previous lines of chemotherapy	1	1 (9)	18 (36)
	2	10 (91)	32 (64)
Response to last treatment before randomisation	First line Platinum-based therapy		
	Progression on treatment (refractory)	0	3/18 (17)
	Primary resistance (< 6 months)	1/1 (100)	9/18 (50)
	Intermediate platinum-sensitive (6–12 months)	0	5/18 (28)
	Non-platinum therapy	0	1/18 (5)
	Second line Platinum-based therapy (for relapse)		
	Progression on treatment (refractory)	0	2/32 (6)
	Primary resistance (< 6 months)	9/10 (90)	24/32 (75)
	Intermediate platinum-sensitive (6–12 months)	0	1/32 (3)
	Non-platinum therapy	1/10 (10)	5/32 (16)

### Dose-limiting toxicity and treatment modifications

To determine the maximum tolerated dose of oral pazopanib as a combination partner of topotecan, 38 documented chemotherapy cycles were evaluated in phase I (Table 2). The dose-escalation study started with 400 mg pazopanib daily for the first three participants. At dose level two administered dose of pazopanib increased to 600 mg daily, one patient continued combination for six cycles after early dose reduction of pazopanib, but two others developed dose limiting haematological and non-haematological toxicities despite dose reduction. For this reason, the study protocol required to continue study, enrolling additional participants to the previous dose level one. Finally, five patients were recruited into this level, one with premature therapy discontinuation (after first cycle) and one other without start of study treatment at all (later died on recurrence). In conclusion, 8 patients were recruited to dose level 1 and 3 patients treated at dose level 2 were comprised for determination of the MTD of pazopanib to 400 mg daily.

**Table 2:** Characteristics of the treatment cohorts

Patient’s characteristics, *n* (%)	Phase I	Phase II
Registered	11	50
Assessable for toxicity/efficacy evaluation	10/0	50/50
Patient’s enrollment and treatment algorithm		
Completed 6 cycles of chemotherapy,* n* (%)	3 (30)	12 (24)
Chemotherapy cycles evaluated (all, median, range)	38 (3; 0–6)	174 (3; 1–6)
Entered maintenance,* n* (%)	2 (20)	9 (18)
Maintenance cycles evaluated (all, median, range)	52 (26, 49 and 3)	43 (3; 1–15)
Phase I, dose level one (*n*)	8	
received no study treatment	1	–
dose reduction / treatment delay	2 / 30	–
premature discontinuation of therapy	5	–
Phase I, dose level two (*n*)	3	–
Dose reduction/treatment delay	1/1	–
Premature discontinuation due to DLT	2	–
Phase II (*n*)	–	50
Dose reduction/treatment delay	–	7 / 172
Premature discontinuation	–	38
Entered Maintenance Treatment,* n* (%)	2 (20)	9 (18)
Dose reduction / treatment delay	1	2/2
Premature discontinuation	1	–

The summarized analysis of the dose escalation phase I included 10 evaluable patients, considering 3 of them who received all planned 6 cycles of pazopanib with topotecan without premature treatment discontinuation (2 at dose level 1 and one at dose level 2) and seven patients who have stopped the combination either due to recurrence (3 after the third and one after the fourth cycle), due to DLT (one with pulmonary embolism and the second one due to prolonged severe neutropenia and thrombocytopenia after third cycle), or due to own request resulting of prolonged haematological adverse events (AE’s) and hepatotoxicity. Two patients of the phase I entered the maintenance part receiving 52 treatment cycles with pazopanib (Table 2).

Treatment delay with interruptions of the chemotherapy courses during the dose escalation study were most common under topotecan (68%) and rarely caused by pazopanib (24%). Altogether 35 dose modifications of topotecan have been reported, 58% of them caused by haematological toxicity like thrombocytopenia, while further nine modifications were caused by pazopanib. Interestingly the only one patient who received all planned six cycles of topotecan and pazopanib at dose level 2 and developed consequently haematological and non-haematological toxicities, switched after first cycle to reduced dose of 400 mg pazopanib and continued it for 192 weeks (49 cycles). For this patient five treatment delays, mostly caused by an isolated gamma-glutamyl transferase (GGT) increase were documented. No dose reductions in the phase I maintenance were observed.

The recommended dose for the phase II of the TOPAZ trial was pazopanib 400 mg daily and topotecan 4 mg/m^2^ on days 1, 8, 15 of a 28-day cycle. In total 174 cycles of chemotherapy with a median of three courses (range 1–6) were administered (Table 2). For the safety and efficacy evaluations the median follow-up was 7.7 months (range 1.2 to 36.5 months). Twelve out of 50 enrolled patients (24%) received all intended 6 courses of topotecan and pazopanib. 21 patients (42%) discontinued treatment because of cancer recurrence (one death), 7 due to non-haematological and 6 due to haematological side effects. Protocol deviations (*n* = 3) and patient decision (*n* = 1) were other reasons for previous treatment stop. Only nine patients (18%) entered the study maintenance, receiving in total 42 cycles (median 3, range 1–15 cycles) of pazopanib treatment.

The most common reason for treatment delay during the phase II chemotherapy resulted due to haematologic AE’s under topotecan (115 of 174 cycles, 66%), while pazopanib caused 57 (33%) of all treatment interruptions. During the six courses of chemotherapy 164 dose modifications for topotecan were documented, mostly due to haematological events (65%), followed by non-hematologic events (8%) or patient’s preference (6%). Pazopanib caused 87 dose modifications during this study phase, mostly due to haematological events (72%). During the maintenance only for two patients’ treatment interruption of pazopanib was documented, not due to toxicity reasons, but several times on patient’s request. For two other participants dose reduction was necessary due to non-haematological events (lever enzymes increase or general condition), after that one of both patients continued with pazopanib maintenance for overall 60 weeks without course delay.

### Safety

For the safety analysis the ITT-cohort comprised of sixty patients–10 from the phase I and 50 from the phase II parts, who all received at least one cycle of study treatment. Altogether no episodes of severe organ failure or treatment related deaths were documented in the study.

Within the dose escalation phase I, in total 272 possibly or probably related to treatment adverse events (AE’s) were calculated per cycle and patient and classified according to the NCI-CTCAE 4.0 (Table 3). Most clinically relevant AE’s (*n* = 159) were associated with pazopanib (58.5%) and other 113 (41.5%) with topotecan treatment. During the dose escalation 59 severe AE’s of NCI-CTCAE grade 3 or 4 were registered—30 within the dose level one and 29 within the dose level two group. Leucopenia (25%), neutropenia (22%) and thrombocytopenia (18.6%) were the most severe haematological AE’s, resulting in DLT. Liver function toxicity of grade 3/4 was commonly presented with 20% by increased alkaline phosphatase (AP) and/or gamma-glutamyl transferase (GGT) denoting a cholestatic damage or with hepatocellular damage (13.6%) measured by increased alanine transaminase (ALT) and/or aspartate transaminase (AST). Interestingly anaemia was exceptionally presented by mild grade 1/2 reactions. Hypertonia and impaired coagulation, both each 3.4% were rarely. Determination of DLT probably resulted during pazopanib treatment, documented in two patients with severe AE’s of NCI-CTCAE grade 4 at dose level two–one with pulmonary embolism and neutropenia and the second patient with prolonged severe neutropenia and thrombocytopenia, which aggravated in both cases to premature discontinuation of treatment. The toxicity evaluation of the phase I maintenance comprised data of the two patients with manifestation of an occasional increase of ALT and GGT, who even in grade 3 does not resulted in dose modification or treatment discontinuation of pazopanib. Six serious adverse events (SAE) were reported for the dose escalation part at all, but only pulmonary embolism and increase of alanine aminotransferase (ALT) were considered as probably or possibly related to pazopanib.

**Table 3:** Toxicities of interest within the cohorts of the phase I and phase II trial

Patient’s characteristics, *n* (%)		Phase I		Phase II	
Registered		11		50	
Assessable for toxicity/efficacy evaluation		10/0		50/50	
Treatment-emergent Adverse Events (TEAE)					
Patients with ≥ one treatment-emergent AE		9		47	
AE’s calculated per cycle and patient (all grades, n)		272		856	
AE’s Grade 3/4 (phase I, separate dose level 1/2, n)		59 (30/29)		203	
Haematological AE’s		all grades grade 3/4		all grades grade 3/4	
Anemia		24 (8.8)	2 (3.4)	107 (12.5)	15 (7.4)
Leucopenia		40 (14.7)	15 (25.4)	89 (10.4)	18 (8.9)
Neutropenia		28 (10.3)	13 (22)	105 (12.3)	42 (20.7)
Thrombocytopenia		55 (22.2)	11 (18.6)	114 (13.3)	32 (15.8)
Non-haematological AE’s		All grades	Grade 3/4	All grades	Grade 3/4
Liver	ALT/AST	44 (16.2)	8 (13.6)	67 (7.8)	10 (4.9)
	AP/GGT	43 (15.8)	12 (20.3)	80 (9.4)	29 (14.3)
	Bilirubin (hyperbilirubinemia)	10 (3.7)	1 (1.7)	16 (1.9)	5 (2.5)
Renal	Creatinine increase	13 (4.8)	0	18 (2.1)	0
	Proteinuria	2 (0.7)	0	15 (1.8)	1 (0.5)
Cardiac	Hypertonia	9 (3.3)	2 (3.4)	28 (3.3)	8 (3.9)
Coagulation	Thromboembolic events	4 (1.5)	2 (3.4)	9 (1.1)	2 (1)
	Prolonged aPTT, TTP, other	7 (2.6)	1 (1.7)	10 (1.2)	2 (1)
General	Nausea/Vomiting	27 (9.9)	0	84 (9.8)	9 (4.4)
	Diarrhea/Constipation	14 (5.1)	0	59 (6.9)	3 (1.5)
	Abdominal pain	17 (6.3)	1 (1.7)	55 (6.4)	2 (1)
	Fatigue	20 (7.4)	0	46 (5.4)	8 (3.9)
	Electrolyte disorders	14 (5.1)	0	34 (4)	11 (5.4)
Dermatological	Rash acneiform/dry skin	2 (0.7)	0	19 (2.2)	0
	Mucositis	3 (1.1)	1 (1.7)	18 (2.1)	1 (0.5)
Neurological	Peripheral neuropathy	12 (4.4)	0	28 (3.3)	2 (1)
Serious adverse events (SAE’s)		6		45	

In total 856 AE’s were identified during the phase II part of the study per cycle and patient as possibly or probably related to study treatment, where 53% (*n* = 453) were associated to pazopanib and 47% (*n* = 403) were caused by topotecan (Table 3). Within all registered AE’s 203 were of NCI-CTCAE grade 3 or 4. Neutropenia (20.7%) and thrombocytopenia (15.8%) were the most common severe haematological toxicities followed by leucopenia (8.9%) and anaemia (7.4%). The most common grade 3/4 non-haematological toxicities resulted mainly from impaired liver function and presented as increase of the cholestatic parameters AP and GGT (14.3%), the hepatocellular enzymes ALT and AST (4.9%), and hyperbilirubinemia (2.5%). Other severe and clinically relevant AE’s included electrolyte disorders (5.4%), nausea/vomiting (4.4%), hypertonia (3.9%) and fatigue (3.9%). The safety analysis of the phase II maintenance comprised data of nine patients with manifestation of different profile of severe AE’s of NCI-CTCAE grade 3 or 4 like: aggravation of general condition, vomiting, cough, dyspnoea, ascites, GGT-increase or subileus, all of them even in grade 3/4 does not resulted in dose modification or treatment discontinuation of pazopanib. 45 SAE’s occurred under the combination treatment within the phase II part, where 20 were classified as possibly treatment-associated, but none of these caused severe organ failure or treatment related deaths. Additionally, 9 SAE’s were documented during the phase II pazopanib maintenance. Whitin these SAE’s general physical health deterioration (*n* = 2), dyspnoea (*n* = 2) and bowel obstruction (*n* = 2) were most common, but none of this was associated with pazopanib treatment. Altogether, the overall toxicities during the phase II part of the TOPAZ trial were mostly temporary and good manageable.

### Efficacy

As defined per protocol only participants treated within the TOPAZ phase II study were included into the efficacy evaluation. Median follow-up time (i.e. time from study registration to disease progression, last contact/visit or death) was 7.7 months with a range of 1.2 to 36.5 months (Table 4). Within the treatment period 38 of the 50 patients developed disease progression and dropped out of further study treatment. At data cut-off (i.e. 12 months after last visit of last patient) 41 patients had died from their cancer.

**Table 4:** Summary of response status (*n* = 50)

Patient’s assessable for response evaluation*	*n*(%)
Registered	50
Assessable for evaluation	39
Median follow-up time (months, range)	7.7 (1.2–36.5)
Response status	
Progressive disease (PD)	5 (10)
Stable disease (SD)	26 (52)
Partial response (PR)	7 (14)
Complete response (CR)	1 (2)
Overall response rate (CR + PR)	8 (16)
Clinical benefit rate (CR + PR + SD)	34 (68)

^*^only patients of the phase II cohort were assessable for response evaluation

A total of 39 patients were assessable for response evaluation. The observed clinical benefit rate (CR, PR and SD) was 68% (34 of 50 patients), where disease stabilisation (SD) was obtained for 26 patients (52%). This study showed an overall response rate of 16% (8 of 50 patients), with 2% presented with CR (*n* = 1), 14% with PR (*n* = 7) and a median duration of response of 120.5 days (95% CI 28–195 days) (table 4).

**Fig. 2:**
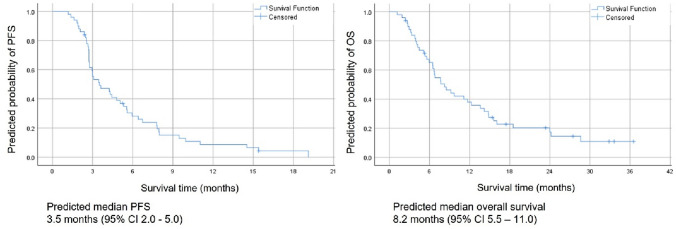
Kaplan–Meier plots for Progressive and Overall survival

The median progression-free survival observed within all phase II participants was 3.5 months (95% CI: 2.0—5.0). The median overall survival was 8.2 months (95% CI: 5.5–11.0) (Fig. 2). Patients with first recurrence (2nd-line cohort, *n* = 18, 36%) demonstrated median progression-free survival of 4.2 months (95% CI 2.5–6.0) and overall survival of 7.7 months (95% CI: 4.5–10.9). Patients with second recurrence (3rd-line cohort, *n* = 32, 64%) demonstrated progression-free survival of 3.1 months (95% CI 2.2–4.0), while the median overall survival was 8.2 months (95% CI: 2.2–14.3).

### Quality of life

The quality of life was measured according to the questionnaires EORTC-QLQ and OVAR28. At least one quality of life (QoL) questionnaire was completed by 5 (50%) of 10 patients in the phase I part and 37 (54%) of the 50 participants treated within the phase II participants at baseline. Statistically significant worsening was observed for physical functioning, role functioning, and emotional functioning, nausea/vomiting and diarrhoea. Global quality of life was worsening, especially from pre chemotherapy (baseline) until after the end of chemotherapy without statistical significance.

## Discussion

This phase I/II study investigated the combination of topotecan with the oral multi-targeted tyrosine kinase inhibitor pazopanib for the treatment of patients with platinum-resistant recurrent ovarian cancer. Based on the safety dose-escalation assessment we determined 400 mg/day pazopanib, as maximum tolerated dose, to be feasible and safe in the simultaneous combination with weekly topotecan followed by maintenance treatment. We were able to demonstrate a high adherence to study treatment, with only two interruptions due to patients’ preference. But as expected for patients in a 2nd and 3rd-line treatment, only 25% received six cycles of chemotherapy (Hanker et al. 2012). The major reasons for treatment discontinuation were progressive disease (42%) and toxicity (26%). As management of recurrent ovarian cancer is a challenging issue our results fit well with data of many prospective randomized trials showing that especially patients with platinum-resistant relapse usually receive not more than three cycles of chemotherapy till development of early disease progression (Hanker et al. 2012; Sehouli et al. 2011; Chekerov et al. 2015).

The summarized analyses within both—dose-finding and safety cohorts demonstrated that most relevant treatment related AE’s of grade 3–4 resulted from the accumulation of bone-marrow and liver toxicities, which finally determined the DLT of pazopanib. Leucopenia (25%), neutropenia (22%) and thrombocytopenia (19%), but also cholestatic (20%) and hepatocellular damage (15%) were identified to be the most clinical important treatment related AE’s. It is reasonable to assume that pazopanib plays a substantial role behind the spectrum of evaluated toxicity, even if severity of some adverse events could also be explained with accumulation of topotecan side-effects (Chekerov et al. 2018; Sehouli et al. 2011; Gordon et al. 2004). These findings are consistently with results of previously reported trials incorporating pazopanib into the adjuvant and recurrent treatment setting of ovarian cancer (Bois et al. 2014; Pignata et al. 2015). Considering the clinical impact of a positive therapeutic index as an essential step of managing relapsed disease, taking into account safety, tolerability and quality of life, our study moreover did not provide data on new severe cumulative toxicity, which could cause a severe organ failure or treatment-related deaths.

The question on the wright way to integrate targeted agents into current treatment of recurrent ovarian cancer remains one of the greatest challenges for gynaecologic oncologists (Colombo 2019; Markman et al. 2010). Although clinical efforts made the integration of anti-angiogenic and PARP inhibitors in the first line treatment possible, patients with recurrent disease still have only limited treatment options and commonly not benefit from scientific progress (Hanker et al. 2012). Thus investigation of the anti-tumoral activity of oral vascular endothelial growth factor (VEGF) and tyrosine kinase inhibitors like pazopanib has long been on the focus of potential clinical interest (Pujade-Lauraine et al. 2014; Chase et al. 2017; Monk et al. 2014; Hurwitz et al. 2009). Two prospective studies, focusing on the recurrent patient’s cohort where platinum might not be the best option, investigated the role of pazopanib more thoroughly. The randomized open-label MITO-11 trial assessed weekly paclitaxel with pazopanib as simultaneous combination in 74 platinum resistant ovarian cancer patients and showed with 6.35 months (95% CI 5.36–11.02) a significantly longer progression-free survival for the combination arm compared to 3.49 months (95% CI 2.01–5.66) for paclitaxel weekly (HR 0.42, 95% CI 0.25–0.69, *p* = 0·0002) [25, 27, 31]. Despite the improvement of OS with 19·1 months for the combination group compared to 13.7 months for paclitaxel weekly there was no statistical relevance (HR 0.60, 95% CI 0.32—1·0.3, one-sided *p* = 0.056) (Pignata et al. 2015). Furthermore, this study demonstrated increased toxicity profile of paclitaxel and pazopanib, with relevant levels of neutropenia, leucopenia, fatigue, hypertonia and increase of ALT and AST. A randomized French study with early relapse during the first year of bevacizumab maintenance 116 patients were assigned to either paclitaxel weekly plus pazopanib 600–800 mg daily or standard paclitaxel weekly 80 mg/m^2^. There was no difference between treatment arms in the 4-month PFS rate (4·9 vs. 5·8 months, respectively) or median overall survival (13·6 vs. 12·9 months, respectively), while this combination resulted again in significant more grade 3/4 AE’s and toxicity-related paclitaxel discontinuations. Pazopanib was discontinued for toxicity in 44% of patients, most commonly for gastrointestinal and vascular events (Joly et al. 2022).

In this context our study combining simultaneously topotecan with pazopanib 400 mg/day demonstrated with 68% an encouraging clinical benefit rate, which could assume some gain in efficacy caused by pazopanib, when compared to the above reports, and was even superior to previously reported activity of established topotecan regiments (Sehouli et al. 2011). Unfortunately, with a median PFS of 3.5 months (95% CI 2.0—5.0) and median overall survival of 8.2 months (95% CI 5.5–11.0), our survival rates were not consistent with the postulated study hypothesis (Pujade-Lauraine et al. 2014; Sehouli et al. 2011). One of the reasons for this effect could be the decision to use topotecan in a weekly schedule, based on the hypothesis of lower toxicity and rational feasibility assessing the combination with daily pazopanib. However, the indications for using topotecan in a weekly schedule is not widely accepted (Sehouli et al. 2011). It remains unclear, if considering our results in the context of the positive results of the MITO-11 study group, whether biological or pharmacokinetic factors such as different dosing schedules, mechanism of action or interactions of the combination partners, or whether differences in the study designs are able to attribute in the consideration to our survival data.

Beyond the studies mentioned above the AGO-OVAR16 trial, a phase 3 study, demonstrated a significant improvement in PFS (progression free survival) for patients with advanced ovarian cancer who have not progressed after first-line platinum therapy and received maintenance therapy with pazopanib versus placebo (5,6 months improvement; HR 0.77; 95% CI: 0.64–0.91; *p* = 0.0021). Although there was no difference observed in OS between pazopanib and placebo, this study underlines the clinical potential of VEGF and TK-inhibitors for the acceptance of innovative maintenance strategies (Edwards et al. 2015). Moreover, the results of the TRIAS trial, a double-blind, randomized phase II study which, for the first time ever, reported a significant survival benefit in both PFS and OS by combining the conventional 5-day schedule of topotecan with sorafenib for the treatment of patients with platinum-resistant ovarian cancer demonstrated clear the clinical role of adding small molecules into oncological treatment strategies (Chekerov et al. 2018). It is therefore not surprisingly why implementation of PARP-inhibitors is currently changing the treatment landscape of primary and relapsed high grade ovarian cancer, marking a paradigm shift towards a multimodal treatment (Colombo 2019).

## Conclusion

We report the first prospective investigation of the combination of the topoisomerase I inhibitor topotecan with the oral multi-targeted tyrosine kinase inhibitor pazopanib for the treatment of patients with platinum-resistant recurrent ovarian cancer. This study demonstrated an acceptable feasibility and tolerability, although the encouraging response benefit rate does not correspond to a substantial survival benefit. Based on these results, we will not pursue the combination of weekly topotecan and pazopanib for relapsed ovarian cancer. Nevertheless, we consider the approach of combining chemotherapy with targeted agents such as VEGF-, TK- or PARP-inhibitors as promising new combination strategy and strongly encourage further clinical trials. The key driver for further innovative treatment development should be biomarker based, despite the fact that until now no one factor is known to predict reliable response to specific cytostatic or antiangiogenetic agents.

## Data Availability

The datasets generated and analysed during the current study are available from the corresponding author on reasonable request.
